# Results from a pilot study on the oral microbiome in children and adolescents with chronic nonbacterial osteomyelitis

**DOI:** 10.1007/s00393-021-01035-x

**Published:** 2021-07-01

**Authors:** Mona Zeus, Stefan Janssen, Hans-Jürgen Laws, Ute Fischer, Arndt Borkhardt, Prasad Thomas Oommen

**Affiliations:** 1 Department of Pediatric Oncology, Hematology and Clinical Immunology, Divison of Pediatric Rheumatology, University Children’s Hospital, Medical Faculty, Heinrich-Heine-University Düsseldorf, Moorenstr. 5, 40225 Düsseldorf, Germany; 2 Algorithmic Bioinformatics, Department of Biology and Chemistry, Justus Liebig University Giessen, Giessen, Germany

**Keywords:** Microbiome, Children, CNO, Autoinflammation, Mikrobiom, Kinder, CNO, Autoinflammation

## Abstract

**Objective:**

To analyze the composition of the oral microbiome in children and adolescents with chronic nonbacterial osteomyelitis (CNO) with respect to age distribution, gender differences, effects of medication, disease activity and the influence of body site.

**Methods:**

The oral microbiome of 20 patients (12 male and 8 female; median age 10.3 years) and 36 controls were examined. Two different sites of the oral cavity were swabbed at two time points. Current medication and disease activity were evaluated and registered at these time points. Samples were subjected to amplicon sequencing of the V4 region of the 16S rRNA gene and Qiime2 was used to calculate alpha and beta diversity for multiple alternative metrics.

**Results:**

On the basis of relative abundances of 975 different suboperational taxonomic units in high throughput next generation sequencing, a significant shift in the composition of the oral microbiome (*p* < 0.02) was observed among patients being treated with different medications. There was a significant difference in bacterial communities between the group aged 3–8 years old and the group aged 9–14 years old. Significant differences were also seen in bacterial colonization on different sites in the oral cavity, but not with respect to gender or disease activity.

**Conclusion:**

We present first data of a pilot study of the oral microbiome in children and adolescents with CNO, a rare autoinflammatory bone disease. Differences of the oral microbiome of diseased children to normal adult controls revealed a possible role of the oral microbiome as modulatory target or biomarker in CNO.

## Abstract (de)

**Ziele:**

Untersuchung des oralen Mikrobioms von Kindern und Jugendlichen mit chronischer nicht bakterieller Osteomyelitis (CNO) in Hinblick auf Alter, Geschlecht, Einfluss von Medikamenten, Krankheitsaktivität und Abstrichort.

**Methoden:**

Das orale Mikrobiom von 20 Patienten (12 männlich und 8 weiblich, mittleres Alter: 10,3 Jahre) und 36 Kontrollen wurde untersucht. Hierfür wurden Abstriche an zwei unterschiedlichen Stellen der Zunge zu zwei Zeitpunkten entnommen. Zudem wurden die aktuelle Medikation und die Krankheitsaktivität zu beiden Zeitpunkten erhoben. Mit den gewonnenen Proben wurde ein Next-Generation-Sequencing Hochdurchsatzverfahren des Amplikonsequenzierens der V4-Region der 16S rRNA durchgeführt. Für die Berechnung der alpha- und beta-Diversitäten mit verschiedenen Metriken wurde Qiime2 verwendet.

**Ergebnisse:**

Relative Häufigkeiten konnten bei 975 „sub-operational taxonomic units“ nachgewiesen werden. Bei unterschiedlicher Medikamenteneinnahme der Patienten zeigte sich ein signifikanter Unterschied des Mikrobioms (*p* < 0,02). Zwischen den Patientengruppen 3–8 Jahre und 9–14 Jahre zeigte sich ebenfalls ein signifikanter Unterschied der bakteriellen Zusammensetzung. An den unterschiedlichen Abstrichorten konnten signifikante Unterschiede nachgewiesen werden. Geschlecht und Krankheitsaktivität ergaben keine signifikanten Unterschiede in der Mikrobiom-Zusammensetzung.

**Schlussfolgerung:**

Diese Pilotstudie zeigt erste Daten zum oralen Mikrobiom bei Kindern und Jugendlichen mit CNO, einer seltenen autoinflammatorischen Knochenerkrankung. Die Unterschiede, welche zwischen dem Mikrobiom erkrankter Kinder und dem der erwachsenen Kontrollgruppe gesehen wurden, weisen auf eine mögliche Rolle des oralen Mikrobioms als potenzieller Biomarker bei chronischer nicht-bakterieller Osteomyelitis hin.

## Background

Chronic nonbacterial osteomyelitis (CNO), also known as chronic recurrent multifocal osteomyelitis (CRMO), is a disease spectrum encompassing a state of monofocal or multifocal autoinflammatory bone lesions, primarily affecting children and adolescents with an age peak between 7 and 12 years [1]. A classical hallmark of the disease is pain due to inflammation of the bone or bone marrow, including vertebrae, often affecting the clavicle and the metaphyses and epiphyses of the humerus, femur and tibia [2, 3]. To a certain extent, CNO can be associated with other inflammatory organ manifestations, such as inflammatory bowel disease, palmoplantar pustulosis, acne conglobata and psoriasis [4, 5].

The CNO is regarded as an autoinflammatory disorder in which the innate immune system is etiopathogenetically involved, leading to a sterile bone inflammation without the presence of autoantibodies or autoreactive T‑cells [6].

In 2014, an interplay between microbial agents and bone erosions in an established CNO mouse model was discussed. In this mouse model, mice spontaneously develop osteomyelitis resembling CNO (*Pstpip2 *^*cmo*^* mice*). A high-fat diet was administered to reduce intestinal *Prevotella* levels in these mice, which was then found to significantly reduce pro-IL-1-beta expression in distant neutrophils. For the first time, diet-associated changes of the microbiome were found to be responsible for regulation of inflammation in an animal model of CNO [7].

Taking these murine findings as a basis, in this study we examined the microbiomes of a cohort of 20 patients with different subtypes of CNO. The study provides initial research on the possible role of the oral microbiome in CNO patients with respect to gender, age, disease activity and the impact of medication.

## Methods

### Patient cohort

Patients were recruited from the Pediatric Rheumatology Outpatient Department of the University Hospital Duesseldorf, Germany between May 2017 and February 2018. With the formal consent of guardians and patients, oral swabs were obtained from 20 patients with a pre-established diagnosis of CNO. For further patient characteristics see Table 1. In addition, a healthy adult control group consisting of 20 female and 16 male subjects was examined.

**Table 1:** Patient characteristics. The data show symptoms which ever occurred during patient’s entire course of disease. Age, symptoms, MRI findings showing the localization of inflammation at disease onset and typical comorbidities exhibit a classical phenotype of children and adolescents in chronic nonbacterial osteomyelitis (numbers in front of brackets are absolute figures, numbers in italics show relative figures).

*Patients (n* *=* *20)*	
Male sex	12 *(0.60)*
Median age (years)	10.3* (3–13)*
3–8 years	5 *(0.25)*
9–14 years	15 *(0.75)*
Disease duration	3.05 years* (0–8)*
*Symptoms at disease onset*	
Pain	19 *(0.95)*
Swelling	9 *(0.45)*
Fever	8 *(0.40)*
Reduced range of motion	5 *(0.25)*
*MRI at disease onset*	
Symmetric lesions	8 *(0.40)*
Hyperostosis	3 *(0.15)*
Sclerosis/fibrosis	5 *(0.25)*
Osteoedema	11* (0.55)*
Soft tissue edema	9 *(0.45)*
Osteolysis	3 *(0.15)*
*Comorbidities*	
Crohn’s disease	1* (0.05)*
Psoriasis	1 *(0.05)*
Arthritis	4* (0.20)*
Acne	2* (0.10)*
Palmoplantar pustolosis	2 *(0.10)*
*Patients (n* *=* *20)*	
*Laboratory parameters at disease onset*	
Elevated CRP > 1 mg/dl	8 *(0.40)*
Median ESR	43 mm/h *(0–140* *mm/h)*
*Medication*	
NSAID	20* (1.00)*
Steroids	6 *(0.30)*
Sulfasalazine	3 *(0.15)*
Methotrexate	4 *(0.20)*
Bisphosphonates	3 *(0.15)*
TNF-alpha inhibitors	3 *(0.15)*
Biopsy	12* (0.60)*
*Localization*	
Multifocal	17* (0.85)*
*Localizations (n* *=* *66*)	
Jaw	2* (0.03)*
Clavicle	3 *(0.05)*
Sternum	2 *(0.03)*
Spine	16 *(0.24)*
Upper extremity	8* (0.12)*
Lower extremity	35 *(0.53)*

*NSAIDS* nonsteroidal anti-inflammatory drugs

The swab was obtained in a standardized manner following at least 30 min without food/beverage consumption and after clearing the oral cavity with water. Swabs were obtained with *Rovers Orgenex Brush RT* (Rovers Medical Devices, Oss, The Netherlands) from the center of the tongue and subsequently with a second brush from the left side of the tongue.

**Fig. 1:**
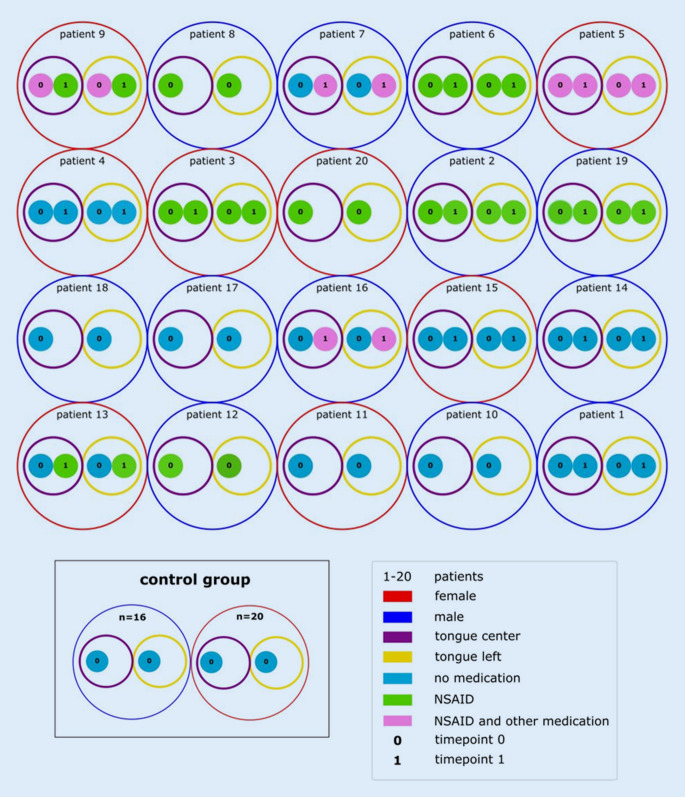
Study design. Oral swabs of 20 patients were taken at time point 0 from the center and the left side of the tongue. Thereof, in 13 patients a second swab was taken from the center and the left side of the tongue at time point 1 (3–6 months after time point 0) and 7 patients did not have a second swab due to loss of follow-up. Furthermore, 36 healthy adults subjects (16 male and 20 female) formed a control group from which oral swabs of the center and the left side of the tongue were obtained at time point 0. At time point 0, 11 patients received no medication, 7 NSAIDs and 2 NSAIDs and other immunomodulatory therapies, such as bisphosphonates or methotrexate. At time point 1, 4 patients received no medication, 6 NSAIDs and 3 NSAIDs and other medication

The timing of the swab acquisition was random and independent of disease activity. A second swab was obtained from 13 patients 3–6 months later. See Fig. 1 for the study design.

After obtaining the swab, brushes were deposited in Safe-Lock 1.5 ml Eppendorf tubes which were prefilled with 500 µl ThinPrep Cytolyt solution.

The following blood values were obtained in order to monitor disease activity and ongoing therapy: complete blood count, calcium, phosphate, alkaline phosphatase, ALT, AST, gamma-glutamyl transferase, creatinine, urea, C‑reactive protein, ferritin, ESR, serum amyloid A, IL‑6, and 25-hydroxyvitamin D.

Medical history, current complaints and medication, as well as a thorough clinical examination including joint count were performed and documented for each patient.

Patient charts were analyzed retrospectively, collecting the following variables for each patient: gender, age at disease onset, symptoms (pain, impaired range of motion, general health status, fever, local signs of inflammation), time to remission, associated comorbidities (Crohn’s disease, psoriasis, arthritis, acne, palmoplantar pustulosis), disease pattern (vertebral, clavicular, sternal type; unifocal or multifocal type), medication (NSAID, steroids, sulfasalazine, methotrexate, bisphosphonates, TNF-alpha blocking agents), MRI results (symmetric lesions, hyperostosis, sclerosis, fibrosis, bone marrow edema, osteolysis), and histological findings.

The study and patient information forms were approved by the local ethics committee of the Medical Faculty, Heinrich-Heine-University (study no. 5828R, registry-ID 2016122195).

### 16S ribosomal RNA (16S) sequencing and analysis

#### DNA extraction.

Oral swab samples were collected from each patient at the two indicated time points and DNA was isolated using the QIAamp Blood Kit (Qiagen) following the pathogen detection protocol.

#### Targeted 16S V4 region sequencing.

Following the Earth Microbiome Project protocols [8], we targeted and amplified the V4 region of the 16S gene by PCR using barcoded primers. V4 paired-end sequencing was performed using a MiSeq (Illumina, La Jolla, CA, USA) according to manufacturer’s protocols.

#### Sequencing data processing and quality control.

Raw V4 sequence reads were demultiplexed using Illumina’s bcl2fastq 2.19.0.316 software. Primers were trimmed via cutadapt 1.18, uploaded to Qiita, a web-based microbiome comparison platform, and quality controlled using the defaults. Forward reads were trimmed to the first 150 nucleotides. The primary feature table was generated using Deblur 1.1.0. Taxonomy assignment was done via the Qiime2 [9] feature classifier plug-in version 2019.1 against Greengenes version 13.8 99% sequence identity OTUs from the 515F/806R region of the sequences. Bacterial features with assigned taxonomy containing the labels c__Chloroplast or f__mitochondria were considered to be of host or plant origin and removed from the feature table prior to rarefaction. Differential abundance analysis was performed via discrete FDR [10] as implemented in Calour, which represents an interactive, microbiome-centric analysis tool.

### Statistics

In order to compute phylogenetic diversity distances, unique V4 sequence fragments were inserted into the reference phylogenetic tree of Greengenes version 13.8 99% sequence identity OTUs [11]. The resulting tree was used to compute weighted and unweighted UniFrac beta distances and Faith’s PD alpha diversity. Rarefaction curves for all samples were computed via Faith’s PD and an optimal rarefaction depth of 30,000 reads per sample was chosen manually, resulting in 66 samples with suitable read counts, totalling 1638 bacterial features.

PIRCUSt was used to predict the meta-transcriptome and BugBase to obtain high-level phenotypes from an OTU table. To generate this table, unrarefied trimmed V4 reads were subjected to closed reference picking via SortMeRNA against Greengenes version 13.8 97% sequence identity OTUs through Qiime2 in Qiita. Nonphylogenetic metrics on predicted feature tables were used for alpha (Shannon, observed OTUs) and beta diversity (Bray-Curtis) calculation.

Differences in community composition for groups of samples (i.e. beta diversity) were assessed via pairwise Permanova tests with 999 permutations as implemented using scikit-bio 0.5.5. Comparison of alpha diversity values and changes in beta diversity was done using Mann-Whitney tests as implemented in scipy 1.1.0.

An NCBI Blast was used to assign taxonomic labels for selected features. Significance was assessed via Mann-Whitney test.

## Results

### Baseline and disease characteristics

In this study 20 patients were evaluated (12 male, 8 female) with a median age of 10.3 years (3–13 years) and 13/20 patients were examined at 2 time points. Moreover, oral swabs of 36 healthy subjects (16 male and 20 female) were taken at time point 0.

At disease onset, 95% of all patients had pain as a leading symptom, swelling of the disease site was reported in 9 patients, reduced range of motion was present in 8 patients, while 5 patients had fever. With respect to disease sites, 17 patients showed a multifocal lesion pattern, with the spine and lower extremities being the most common sites. Laboratory studies showed a normal blood count in 19 patients. In 8 patients, there was an elevation of C‑reactive protein, while the median ESR was 43 mm/h.

The following medications were taken: all 20 patients received nonsteroidal anti-inflammatory drugs (NSAIDs), steroids were used in 6 patients, 4 patients received methotrexate, 2 patients were treated with TNF inhibitors and 3 patients received bisphosphonates.

### Microbiome studies

#### Time points 0 and 1

A time course-specific analysis was not conducted as the number of complete time series was too low. Instead, paired samples from the same individual were either stratified by time point or both time points were combined for subsequent specific analyses.

#### Role of age and sex

Based on patient age, two subgroups were formed and analyzed: 3–8 years and 9–14 years (no patient was older than 14 years). The grouping was chosen in order to be able to differentiate between patients in the typical age of disease onset (9–14 years) and a rather unusual age group (3–8 years).

**Fig. 2:**
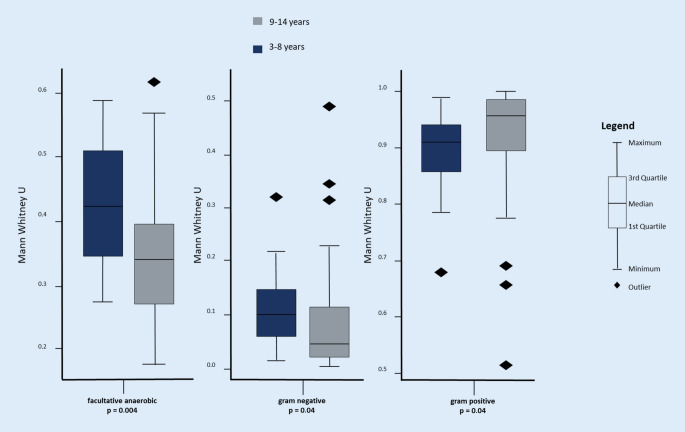
Microbial phenotypic differences by patient age group. Patients aged 3–8 years showed significantly more facultative anaerobic und gram-negative bacteria and less gram-positive bacteria than patients aged 9–14 years, based on BugBase predictions from 16S read counts (two-sided Mann-Whitney test)

Analysis of beta diversity revealed a significant difference between the age groups (Bray-Curtis reaches *p* : 0.001). Taxonomically, the group aged 3–8 years (*n* = 18) showed more gram-negative and facultatively anaerobic bacteria (*p* : 0.004), while more gram-positive bacteria were seen in the group aged 9–14 years (*n* = 48). See Fig. 2 for further information. There was no gender-specific change in microbiome, in either alpha or beta diversity (*p* : 0.16).

#### Body site

Examination of the oral microbiome was performed as described above. On the basis of beta diversity, a significant difference in microbiota was seen between the left side of the tongue and the tongue center (*p* : 0.02). Taxonomically, on the left side of the tongue more *Megasphaera micronuciformis, Haemophilus sputorum, paraphrohaemolyticus, para, Lachnoanaerobaculum orale, Corynebacterium terpenotabidum *and* C. argentoratense *was found, while, in the center, a predominance of *Prevotella histicola, Fusobacterium periodonticum, Porphyromonas pasteri, Solobacterium moorei, Mogibacterium pumilum, neglectum, Actinomyces graevenitzii, A.naeslundii, A.oris *and* A.viscosus *were detected. See Table 2.

**Table 2:** Identified bacteria at the different swab locations. Various species of bacteria were found applying NCBI Blast

	Tongue left	Tongue center
Gram negative	Megasphaera micronuciformis	Prevotella histicola
	Haemophilus sputorum, paraphrohaemolyticus	Fusobacterium periodonticum
		Porphyromonas pasteri
Gram positive	Lachnoanaerobaculum orale	Solobacterium moorei
	Corynebacterium terpenotabidum, argentoratense	Mogibacterium pumilum, neglectum
		Actinomyces graevenitzii, naeslundii, oris, viscosus

#### Disease activity

In order to examine the impact of disease activity on the microbiome, patients with and without clinical symptoms were compared. Due to the small number of patients with disease activity at the time of sampling, the detected difference in beta-diversity calculated on the basis of Bray-Curtis metric barely reached significance (*p* : 0.05). Disease activity was measured by combining subjective pain described by patient/guardian with the visual analogue scale (VAS), physical examinations performed by physicians and laboratory values of inflammation (CRP and ESR).

#### Medication and change in microbiome

As patients’ treatment regimens differed substantially, different treatment groups were formed: I. NSAID only; II. NSAID + (encompassing all combination therapies, such as methotrexate, sulfasalazine, TNF-blocking agents and bisphosphonates); III. no therapy. At time point 0 group I consisted of 7 patients, 2 patients were listed in group II and 11 patients were included in group III. At time point 1 the groups were built as follows: 6 patients (group I), 3 patients (group II) and 4 patients (group III).

**Fig. 3:**
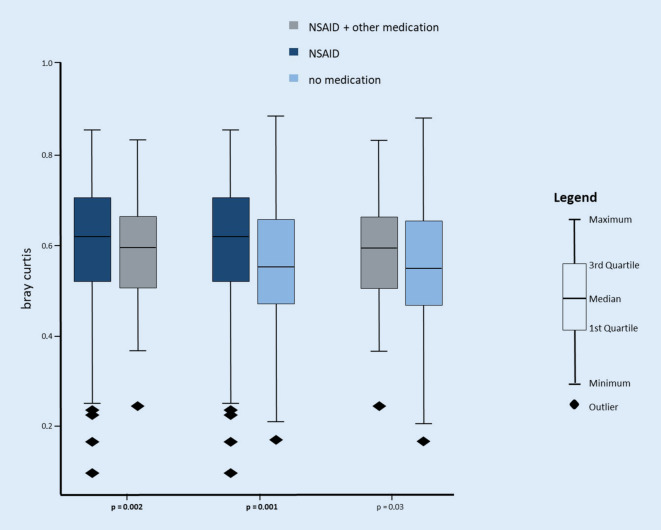
Impact of medication (Bray-Curtis metric). No medication *n* = 30, NSAID *n* = 26, NSAID + further med. *n* = 10. Patients with NSAIDs showed a significantly different microbiome compared to unmedicated patients and compared to patients who took NSAIDs plus further medication

Comparisons of groups I and II and groups I and III revealed a clear and significant change of the oral microbiome in patients who were treated with either NSAID or NSAID + as opposed to patients who were without treatment. Those who were unmedicated had more abundant aerobic and less abundant anaerobic bacteria, in contrast to patients who were treated with NSAIDS. This effect was clearly observed using different prediction methods (Bray-Curtis on PICRUSt predicted Rfam and COG tables). See Fig. 3.

#### Control group

**Fig. 4:**
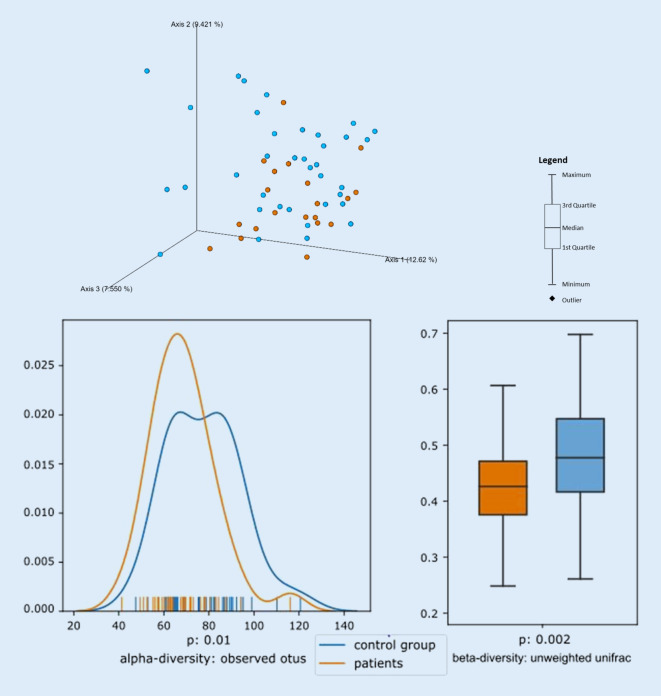
CNO patients have a significantly different oral microbiome compared to healthy controls. Pairwise unweighted UniFrac distances (beta diversity) were computed for all samples from tongue left at the first time point. Distance metric was ordinated via principal coordinates analysis (PCoA) into 3D and visualized via EMPeror. Axes indicate percentage of explained variance. Orange spheres indicate patient samples, while blue spheres represent control group samples. At the left side of the tongue alpha diversity of the metric observed_OTUs revealed a *p*-value of 0.01 while beta diversity of the metric unweighted unifrac revealed a *p*-value of 0.002. Patients *n* = 20, control group *n* = 36

**Fig. 5:**
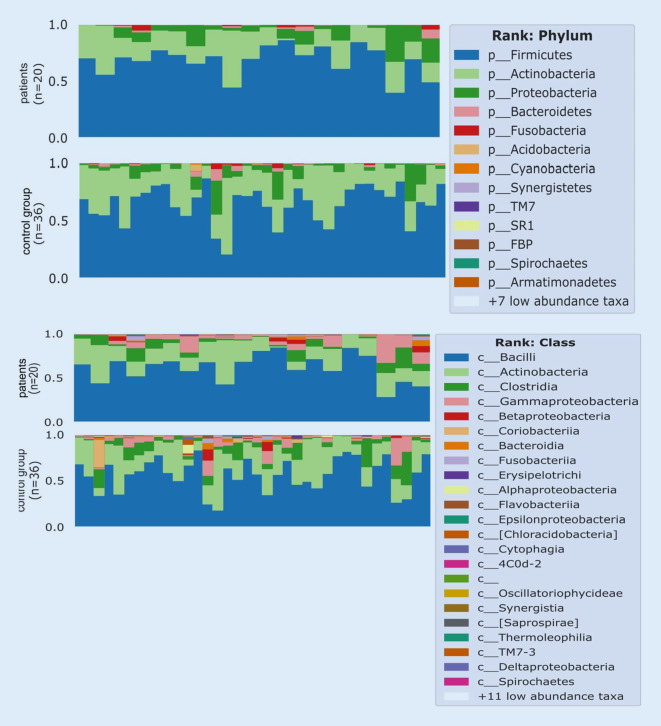
Phylum and class abundance plots left side of the tongue, timepoint 0. Taxonomy plot for left tongue microbiome samples of first time point from patients (*n* = 20) and control group (*n* = 36). Taxonomy assignment was done via Qiime2’s “feature-classifier” plugin version 2020.2 against Greengenes 13_8 99% OTUs from 515F/806R regions of the observed sequence reads

As a control group, 36 healthy adult subjects (16 male and 20 female) were examined. A significant difference was seen between the patients’ microbiomes and those of the control group. Regarding the left side of the tongue, the determined alpha diversity of the metric observed_otus revealed a *p*-value of 0.01, while the beta diversity of the metric unweighted_unifrac revealed a *p*-value of 0.002 (see Figs. 4 and 5). This pattern was also found in swabs taken from the center of the tongue.

#### Robustness tests—analyzing independence of variables

Correlating the metadata with different variables (localization, disease activity, time point, medication, age, etc) of the 66 samples showed that only medication, swab and time point slightly correlate (0.19), as do medication, swab and age group (0.1). Creating a linear mixed model and performing a forward-step redundancy analysis of the first 10 principle components of Bray-Curtis distances showed that medication, swab, age group and localization significantly correlate with microbial communities, while correlations with sex and disease activity did not reach significance; however, another model, considering only disease activity, identified significant microbial differences between samples from patients with and without disease activity at the first time point.

## Discussion

This research paper can be considered a pilot study, providing initial insights into the oral microbiome in CNO in children and adolescents. A significant and interesting finding was a possible impact of anti-inflammatory and immunomodulatory medication on the composition of the oral microbiome.

We sampled the oral microbiome, while most other microbiome studies in children and adolescents with rheumatic diseases—mostly dealing with juvenile idiopathic arthritis (JIA)—have used stool samples. Despite confounding factors, such as intake of food and beverages or dental health, the oral cavity is (the first) part of the digestive system and therefore a representative source encompassing 50–100 billion bacteria and approximately 700 predominant taxa [12]. The composition of the oral microbiome may also be linked to nonoral diseases [13, 14]. Furthermore, easy access to material and a high return ratio are practical arguments for our choice, especially in the context of rheumatoid arthritis, a frequent subject for microbiome studies [15, 16]. In order to rule out methodological bias, accurate and separate handling of the material and separate wet-lab handling of swabs from different sites was performed, to enhance the quality of the data.

We found that there was a significant difference with respect to the distribution pattern of commensals in the oral cavity. This finding shows that the distribution pattern of microbiota in the oral cavity seems to follow a distinct pattern. This pattern has to be anticipated when conducting studies of the oral microbiome. As most microbiome studies only examine the intestinal microbiome, this study presents unique descriptive data from the oral cavity in CNO. The results of this pilot study emphasize that an accurate research design is required for studies of the oral microbiome, since even small deviations regarding the smear location can alter the data. The differences have been shown before [12, 17, 18] and follow-up studies will further investigate them.

With respect to patient age, there was a significant and interesting difference in microbiota between our subgroups of 3–8 and 9–14 years of age. Whether age-specific environmental factors, such as food intake or host immune system, serve as explanation for this finding is yet to be answered.

Finally, the most important finding was the significant difference in microbiota composition identified when comparing samples from patients who were treated with NSAIDs or NSAIDs plus (+) a disease-modifying antirheumatic drug (DMARD), such as methotrexate, sulfasalazine, prednisone or bisphosphonates. Thus, the use of medication revealed alterations that show that anti-inflammatory and immunomodulating strategies, such as COX‑1 and COX‑2 inhibition, or interference with purine metabolism and downregulation or blockade of proinflammatory cytokines may play a measurable role in the shift of the oral microbiome. Regression analyses would be necessary to further confirm this hypothesis; however, our pilot study only involves a relatively small sample size where these statistical methods are of no further benefit. So in order to further clarify the actual role of the microbiome in this context, larger studies with more patients and more homogeneous cohorts are needed.

No significant differences were seen between male and female patients; however, this may also be attributed to the small sample size.

Altogether, this pilot study provides the first description of the oral microbiome of children and adolescents with CNO. This study adds to the hypothesis that alterations of the microbiome may play a role in CNO and that the oral microbiome may be used for a systematic analysis of microbial changes in autoinflammatory bone disease, helping to better understand the complex interplay of genetics, cytokine dysregulation and environmental factors in the etiopathogenesis of CNO.

As patients were examined independently of disease activity in our study, we examined two different time points in order to detect an individual change of the microbiome at different states of disease activity. The control group consisted of 36 healthy adults, and a significant difference in microbial composition between patients and controls was detected; however, in order to overcome age-dependent differences an age-matched group should be the goal for future trials in order to better verify these findings.

A shortcoming of our study was the small sample size and the heterogeneity with respect to disease pattern (unifocal vs. multifocal). Here, future trials with a greater sample size and a more homogeneous group are needed to further elucidate the actual impact of the microbiome and furthermore draw conclusions regarding different microbial phenotypes.

In the context of juvenile rheumatic diseases, studies dealing with the role of the microbiome in juvenile idiopathic arthritis, the most common rheumatic disease in children and adolescents, have shown interesting results [19, 20]. Recently, a prospective inception cohort of Dutch and Italian JIA patients has supported the hypothesis of gut dysbiosis in JIA. The group showed decreased richness in samples from patients with persistent disease activity compared to healthy controls. A relative increased or decreased abundance of certain species (e.g. *Erysipelotrichaceae, Allobaculum, Faecalibacterium prausnitzii*) was found in patients and controls [21].

Dong et al. presented data on the change of the (intestinal) microbiome in another autoinflammatory disease, systemic juvenile idiopathic arthritis, demonstrating microbial differences between children in two different disease states (active, inactive) and healthy children [22].

## Conclusion

This pilot study of the oral microbiome in children and adolescents with CNO generated data regarding the microbiome’s potential influence on disease activity. Microbial changes between age groups and upon introduction of anti-inflammatory and immunomodulatory medication were seen.

Further studies in bigger, homogeneous collectives are needed to further elucidate the role of the microbiome in the etiopathogenesis of autoinflammatory disease, in order to ultimately tailor therapeutic strategies comprising dietary interventions that may help to achieve a lasting remission.

## Abbreviations

ALT: Alanine aminotransferase
AST: Aspartate aminotransferase
CNO: Chronic non-bacterial osteomyelitis
COG: Clusters of orthologous groups
COX: Cyclooxygenase
CRMO: Chronic recurrent multifocal osteomyelitis
CRP: C‑reactive protein
DMARD: Disease-modifying antirheumatic drug
DNA: Deoxyribonucleic acid
ESR: Erythrocyte sedimentation rate
FDR: False discovery rate
IL‑6: Interleukin 6
JIA: Juvenile idiopathic arthritis
MRI: Magnetic resonance imaging
NCBI: National Center for Biotechnology Information
NSAID: Nonsteroidal anti-inflammatory drug
OTU: Operational taxonomic unit
PICRUST: Phylogenetic investigation of communities by reconstruction of unobserved states
PCR: Polymerase chain reaction
Qiime: Quantitative insights into microbial ecology
16Sr-RNA: 16 Svedberg ribosomal ribonucleic acid
TNF‑α: Tumor necrosis factor alpha
VAS: Visual analogue scale

## References

[1] Jansson AF, Grote V (2011). Nonbacterial osteitis in children: data of a German Incidence Surveillance Study. Acta Paediatr.

[2] Catalano-Pons C, Comte A, Wipff J, Quartier P, Faye A, Gendrel D, Duquesne A, Cimaz R, Job-Deslandre C (2008). Clinical outcome in children with chronic recurrent multifocal osteomyelitis. Rheumatology (Oxford).

[3] Jansson A, Renner ED, Ramser J, Mayer A, Haban M, Meindl A, Grote V, Diebold J, Jansson V, Schneider K, Belohradsky BH (2007). Classification of non-bacterial osteitis: retrospective study of clinical, immunological and genetic aspects in 89 patients. Rheumatology (Oxford).

[4] Beck C, Morbach H, Beer M, Stenzel M, Tappe D, Gattenlohner S, Hofmann U, Raab P, Girschick HJ (2010). Chronic nonbacterial osteomyelitis in childhood: prospective follow-up during the first year of anti-inflammatory treatment. Arthritis Res Ther.

[5] Wipff J, Costantino F, Lemelle I, Pajot C, Duquesne A, Lorrot M, Faye A, Bader-Meunier B, Brochard K, Despert V, Jean S, Grall-Lerosey M, Marot Y, Nouar D, Pagnier A, Quartier P, Job-Deslandre C (2015). A large national cohort of French patients with chronic recurrent multifocal osteitis. Arthritis Rheumatol.

[6] Morbach H, Hedrich CM, Beer M, Girschick HJ (2013). Autoinflammatory bone disorders. Clin Immunol.

[7] Lukens JR, Gurung P, Vogel P, Johnson GR, Carter RA, McGoldrick DJ, Bandi SR, Calabrese CR, Vande Walle L, Lamkanfi M, Kanneganti TD (2014). Dietary modulation of the microbiome affects autoinflammatory disease. Nature.

[8] Gilbert JA, Jansson JK, Knight R (2014). The Earth Microbiome project: successes and aspirations. BMC Biol.

[9] Bolyen E, Rideout JR, Dillon MR, Bokulich NA, Abnet CC (2019). Reproducible, interactive, scalable and extensible microbiome data science using QIIME 2. Nat Biotechnol.

[10] Jiang L, Amir A, Morton JT, Heller R, Arias-Castro E, Knight R (2017). Discrete false-discovery rate improves identification of differentially abundant microbes. mSystems.

[11] Janssen S, McDonald D, Gonzalez A, Navas-Molina JA, Jiang L, Xu ZZ, Winker K, Kado DM, Orwoll E, Manary M, Mirarab S, Knight R (2018). Phylogenetic placement of exact amplicon sequences improves associations with clinical information. mSystems.

[12] Krishnan K, Chen T, Paster BJ (2017). A practical guide to the oral microbiome and its relation to health and disease. Oral Dis.

[13] Ahn J, Chen CY, Hayes RB (2012). Oral microbiome and oral and gastrointestinal cancer risk. Cancer Causes Control.

[14] Docktor MJ, Paster BJ, Abramowicz S, Ingram J, Wang YE, Correll M, Jiang H, Cotton SL, Kokaras AS, Bousvaros A (2012). Alterations in diversity of the oral microbiome in pediatric inflammatory bowel disease. Inflamm Bowel Dis.

[15] Scher JU, Ubeda C, Equinda M, Khanin R, Buischi Y, Viale A, Lipuma L, Attur M, Pillinger MH, Weissmann G, Littman DR, Pamer EG, Bretz WA, Abramson SB (2012). Periodontal disease and the oral microbiota in new-onset rheumatoid arthritis. Arthritis Rheum.

[16] Zhang X, Zhang D, Jia H, Feng Q, Wang D (2015). The oral and gut microbiomes are perturbed in rheumatoid arthritis and partly normalized after treatment. Nat Med.

[17] Liljemark WF, Bloomquist CG, Uhl LA, Schaffer EM, Wolff LF, Pihlstrom BL, Bandt CL (1984). Distribution of oral Haemophilus species in dental plaque from a large adult population. Infect Immun.

[18] Delorme C, Abraham A-L, Renault P, Guédon E (2015). Genomics of Streptococcus salivarius, a major human commensal. Infect Genet Evol.

[19] Hissink Muller P, Meij T, Westedt M, de Groot E, Allaart C, Brinkman D, Schonenberg D, Van den Berg JM, Van Suijlekom-Smit L, Rossum M, Budding A, Cate R (2017). Disturbance of microbial core species in new-onset juvenile idiopathic arthritis. J Pediatr Infect Dis.

[20] Tejesvi MV, Arvonen M, Kangas SM, Keskitalo PL, Pirttilä AM, Karttunen TJ, Vähäsalo P (2016). Faecal microbiome in new-onset juvenile idiopathic arthritis. Eur J Clin Microbiol Infect Dis.

[21] van Dijkhuizen EHP, Del Chierico F, Malattia C, Russo A, Pires Marafon D, Ter Haar NM, Magni-Manzoni S, Vastert SJ, Dallapiccola B, Prakken B, Martini A, De Benedetti F, Putignani L (2019). Microbiome analytics of the gut microbiota in patients with juvenile idiopathic arthritis: a longitudinal observational cohort study. Arthritis Rheumatol.

[22] Dong YQ, Wang W, Li J, Ma MS, Zhong LQ, Wei QJ, Song HM (2019). Characterization of microbiota in systemic-onset juvenile idiopathic arthritis with different disease severities. World J Clin Cases.

